# Multifocal Cerebral Microabscesses Secondary to Staphylococcus aureus Catheter-Related Bloodstream Infection in a Hemodialysis Patient

**DOI:** 10.7759/cureus.96714

**Published:** 2025-11-12

**Authors:** Tamar Kasradze, Tamar Didbaridze, Gvantsa Metskhvarishvili, Irma Tchokhonelidze

**Affiliations:** 1 Nephrology, Tbilisi State Medical University and Ingorokva High Medical Technology University Clinic, Tbilisi, GEO; 2 Internal Medicine, Tbilisi State Medical University, Tbilisi, GEO; 3 Microbiology, The First University Clinic of Tbilisi State Medical University, Tbilisi, GEO

**Keywords:** catheter-related bloodstream infection, central venous catheter (cvc), cerebral microabscesses, hd (hemodialysis), staphylococcus aureus

## Abstract

Staphylococcus aureus is a common cause of catheter-related bloodstream infections (CRBSIs) in patients undergoing hemodialysis, although cerebral microabscesses resulting from septic emboli are exceedingly rare. We describe a 37-year-old woman on chronic hemodialysis through a tunneled central venous catheter who presented with headache, limb weakness, and confusion without fever. Blood cultures confirmed methicillin-sensitive Staphylococcus aureus, and brain MRI demonstrated multifocal diffusion-restricted lesions consistent with microabscesses. Following prompt catheter removal and a six-week course of intravenous antibiotics, the patient achieved full neurological recovery and was transitioned to permanent vascular access. This case highlights the importance of early recognition of unusual neurological manifestations of Staphylococcus aureus CRBSI and the critical role of timely imaging and definitive source control in improving outcomes.

## Introduction

Central venous catheters (CVCs) remain a vital yet challenging vascular access option for patients requiring urgent or long-term hemodialysis. Despite their utility, they are associated with disproportionately high infection rates compared with arteriovenous fistulas (AVFs) or grafts (AVGs) [1,2]. Catheter-related bloodstream infections (CRBSIs) contribute not only to excess morbidity and mortality but may also result in severe metastatic complications such as infective endocarditis, vertebral osteomyelitis, and intracranial abscesses [3,4]. Brain abscesses, although exceedingly rare, carry substantial morbidity and mortality, particularly when diagnosis and intervention are delayed [5].

Staphylococcus aureus is the most commonly implicated pathogen in CRBSIs, well known for its ability to disseminate hematogenously and invade distant tissues, thereby increasing the risk of metastatic infections [6,7]. We present a case of multiple cerebral microabscesses secondary to hematogenous septic emboli from a CRBSI in a patient receiving chronic hemodialysis via a tunneled CVC. In CRBSI, bacteria enter the systemic circulation through the catheter lumen and may seed distant organs, including the brain, where they form multiple infectious foci. These disseminated lesions are referred to as septic emboli; however, they do not represent particulate embolization originating from the catheter, but rather bacteremia with hematogenous spread leading to microabscess formation. This case highlights key diagnostic challenges and therapeutic considerations and underscores the importance of prevention strategies in patients with chronic catheter dependence.

## Case presentation

A 37-year-old woman with kidney failure secondary to hypertensive nephrosclerosis had been receiving hemodialysis through a tunneled right internal jugular CVC for six months. Hemodialysis was initiated with a tunneled catheter because the patient was referred late to nephrology, leaving insufficient time for AVF creation and maturation, and kidney transplantation had already been planned as the intended renal replacement therapy.

The patient presented with a 24-hour history of progressive headache, bilateral lower extremity weakness, dysarthria, disorientation, and decreased level of consciousness. On examination, she was disoriented with dysarthric speech. Lower extremity muscle strength was reduced, while upper extremity strength was preserved. Cranial nerves were intact, reflexes were symmetric, and no sensory deficits or meningeal signs were present. She denied fever, chills, or systemic infectious symptoms prior to admission, and no local signs of catheter-related infection were observed.

**Figure 1:**
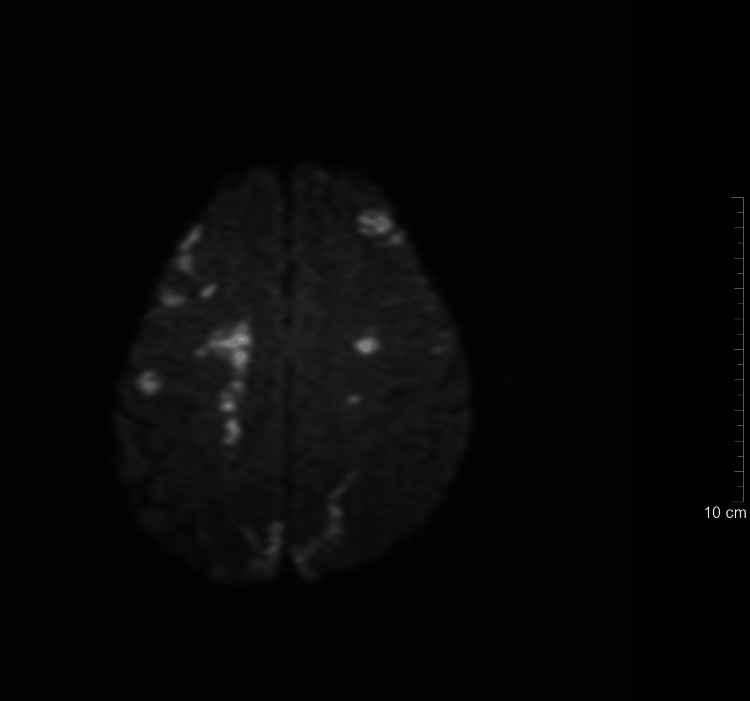
Pretreatment brain MRI showing scattered lesions involving the bilateral subcortical and deep white matter, consistent with hematogenous septic emboli. Diffusion-weighted MRI obtained at admission showing multiple scattered hyperintense lesions in the bilateral frontal and parietal subcortical white matter. The lesions exhibit restricted diffusion, consistent with septic emboli and early cerebral microabscess formation secondary to Staphylococcus aureus catheter-related bloodstream infection.

**Figure 2:**
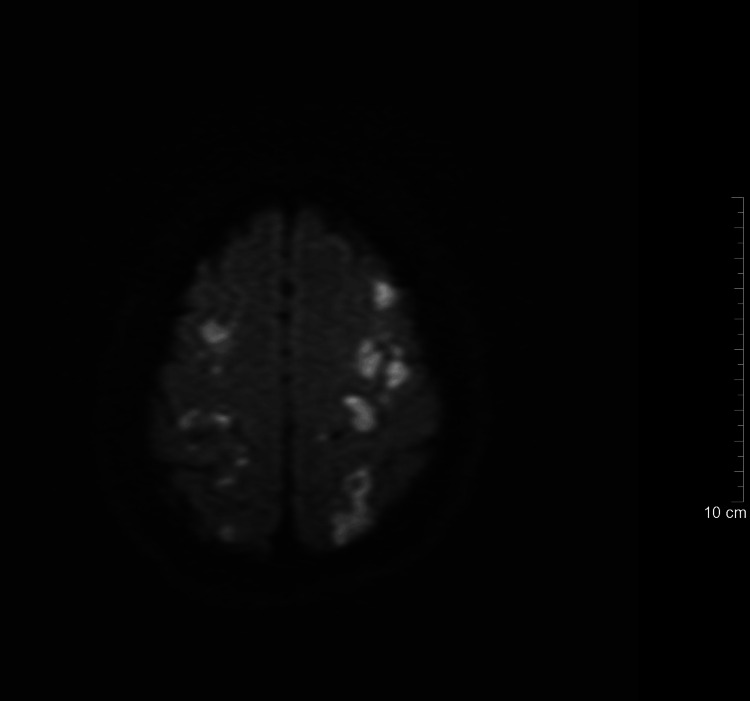
Pretreatmen diffusion-weighted MRI demonstrating multiple hyperintense lesions in the bilateral frontal and parietal lobes, consistent with septic emboli with cerebral microabscess formation. Axial diffusion-weighted MRI obtained at admission demonstrates multiple hyperintense lesions within the bilateral frontal and parietal subcortical white matter, consistent with septic emboli resulting from hematogenous spread of Staphylococcus aureus originating from a tunneled hemodialysis catheter.

Laboratory evaluation showed leukocytosis and elevated C-reactive protein. Cerebrospinal fluid analysis was unremarkable. Paired blood cultures obtained at admission (from the catheter and a peripheral vein) later grew methicillin-sensitive Staphylococcus aureus (MSSA) (Table 1). Final culture and susceptibility results became available on Day 7. Contrast-enhanced brain MRI demonstrated multiple T2/fluid attenuation inversion recovery (FLAIR) hyperintense lesions with restricted diffusion affecting the bilateral semioval centers, subcortical frontal and parietal lobes, cerebellar hemispheres, and the splenium of the corpus callosum, consistent with multiple cerebral microabscesses (Figures 1, 2). Transesophageal echocardiography excluded infective endocarditis.

**Table 1:** Baseline and follow-up laboratory results. Laboratory results demonstrate marked inflammatory response and methicillin-sensitive Staphylococcus aureus (MSSA) bacteremia at presentation. Normalization of leukocyte count and C-reactive protein after one week reflects effective antimicrobial therapy and clinical recovery.

Parameter	Day 1 (At Presentation)	Day 7 (Follow-up)	Reference Range	Interpretation
White Blood Cell count (×10⁹/L)	15.0	7.4	4.0 – 10.0	Elevated on admission, normalized by Day 7
Platelet count (×10⁹/L)	180	—	150 – 400	Within normal limits
Hemoglobin (g/dL)	9.4	—	12.0 – 16.0	Mild anemia
Prothrombin Time (sec)	12	—	11 – 15	Normal
Partial Thromboplastin Time (sec)	29	—	25 – 35	Normal
Thrombin Time (sec)	14	—	13 – 17	Normal
Fibrinogen (g/L)	5.0	—	2.0 – 4.0	Elevated
C-Reactive Protein (mg/L)	180	15	< 5	Markedly elevated, normalized with treatment
Blood Culture	MSSA positive		—	Confirms Staphylococcus aureus bacteremia
Cerebrospinal Fluid	Normal	Normal	—	No meningeal involvement

The CVC was removed, and empiric intravenous vancomycin and meropenem were initiated due to the severity of the patient’s neurological symptoms and concern for possible central nervous system involvement. Meropenem was continued for a total of 14 days during hospitalization, and vancomycin was continued as targeted monotherapy to complete a six-week course. The total duration of antibacterial therapy was six weeks with vancomycin and two weeks with meropenem.

**Figure 3:**
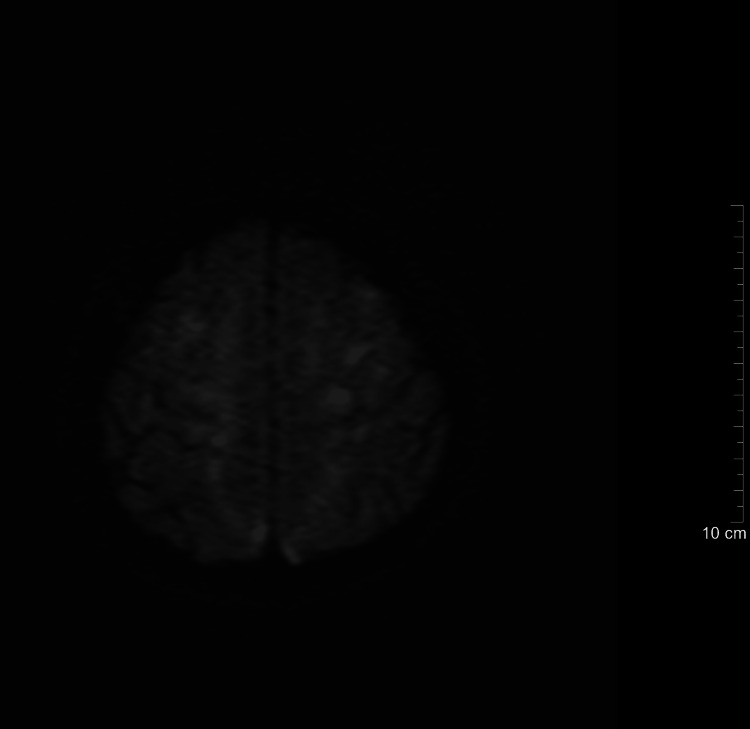
Post-treatment brain MRI (diffusion-weighted imaging, Day 7). This diffusion-weighted MRI obtained seven days after initiation of intravenous antimicrobial therapy shows marked reduction in restricted-diffusion lesions compared to baseline.

**Figure 4:**
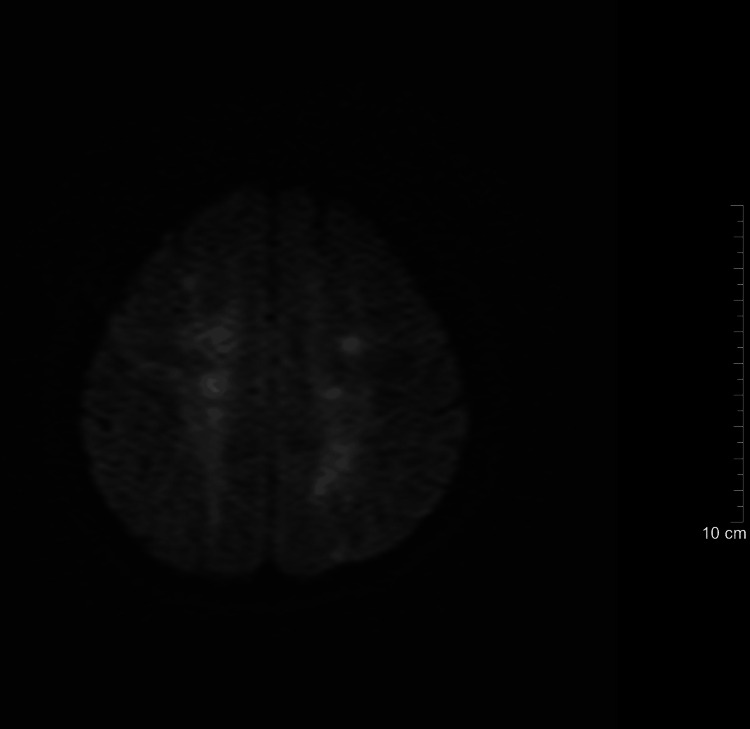
Post-treatment brain MRI (diffusion-weighted imaging, Day 7)

After one week of treatment, neurological deficits resolved, and follow-up MRI demonstrated radiologic improvement (Figures 3, 4). The previously visualized multifocal hyperintense foci - representing septic emboli with microabscess formation - showed a marked decrease in both size and number following catheter removal and targeted antimicrobial therapy.

At the six-month follow-up, the patient remained asymptomatic without recurrent infection or neurologic deficits. She was successfully transitioned to a functioning arteriovenous fistula and subsequently underwent kidney transplantation.

## Discussion

This case highlights the serious potential of dialysis CRBSI to progress from bacteremia to life-threatening cerebral septic emboli and microabscess formation. CRBSI is one of the most significant complications of hemodialysis using CVCs, with Staphylococcus aureus as the predominant pathogen. Its ability to disseminate hematogenously often results in endocarditis, osteomyelitis, or visceral abscesses [8,9]. Among these, cerebral septic emboli complicated by microabscess formation represent rare but life-threatening manifestations arising from the hematogenous spread of infected thrombotic material [10].

Despite advances in neuroimaging and antimicrobial therapy, septic cerebral emboli complicated by microabscesses carry substantial morbidity and mortality, reported between 10% and 40%, particularly in cases of Staphylococcus aureus bacteremia [11].

Several factors increase the risk of CRBSI in hemodialysis patients. Biofilm formation on catheter surfaces protects microorganisms from host immunity and antimicrobials, enabling persistent bacteremia [12]. Additionally, uremia, diabetes, and chronic systemic inflammation impair immune defenses, further predisposing patients to invasive infections [13,14]. In this patient, prolonged catheter dependence likely facilitated hematogenous spread of S. aureus to the central nervous system.

The clinical presentation was atypical: progressive neurological decline - including bilateral motor deficits, dysarthria, disorientation, and altered consciousness - occurred without fever. Such features could easily have been misattributed to metabolic encephalopathy or uremia, which are common in advanced kidney disease. However, inflammatory markers and paired positive blood cultures for MSSA established catheter-associated sepsis. Importantly, neuroimaging demonstrated multifocal diffusion-restricted lesions consistent with septic emboli progressing to microabscesses, with a distribution pattern strongly suggestive of hematogenous seeding from the CRBSI.

Neuroimaging plays a pivotal role in early diagnosis. Diffusion-weighted MRI (DWI) is highly sensitive for detecting microabscesses and septic emboli, often revealing abnormalities before overt neurological symptoms develop [15]. Multifocal punctate hyperintense lesions on DWI within the subcortical and deep white matter - particularly in the frontal and parietal lobes - are characteristic of septic emboli complicated by microabscess formation [16]. Over time, these lesions may progress to ring-enhancing abscesses with central diffusion restriction and surrounding vasogenic edema, reflecting liquefaction and capsule formation.

Management required a multidisciplinary approach. Prompt catheter removal eliminated the nidus of infection, while empirical broad-spectrum therapy was rapidly initiated and then tailored to culture results. A six-week intravenous course resulted in full recovery and resolution of brain lesions. Transitioning to an AVF further reduced the risk of future CRBSI and reinforced the importance of creating durable vascular access for long-term hemodialysis patients [8,9].

## Conclusions

From a broader perspective, this case highlights several key clinical principles. First, CRBSI caused by S. aureus must always prompt a thorough evaluation for potential metastatic complications, including involvement of the central nervous system. Second, neurological deterioration in dialysis patients should not be hastily attributed to metabolic imbalances; infectious causes must be actively ruled out. Third, prompt neuroimaging is essential for accurate diagnosis and effective treatment planning. Lastly, preventive strategies - particularly the early creation of durable arteriovenous access - remain vital to decreasing dependence on CVCs and reducing the burden of CRBSI in this population.
